# Endoscopic closure of gastric tube perforations with titanium clips: a four-case report

**DOI:** 10.1186/s12957-015-0434-8

**Published:** 2015-02-07

**Authors:** Xianghong Zhan, Bin Wang, Dongmei Di, Yun Zhuang, Xiaoying Zhang, Jianping Chen

**Affiliations:** Department of Cardiothoracic Surgery, the Third Affiliated Hospital of Soochow University, 185 Juqian Street, Changzhou, 213003 China; Department of Digestive Medicine, the Third Affiliated Hospital of Soochow University, 185 Juqian Street, Changzhou, 213003 China

**Keywords:** Esophago-cardial carcinoma, Esophagogastrostomy, Gastric tube perforation, Endoscopic clip, Gastroscope

## Abstract

Perforation of a gastric tube is a rare yet lethal complication after esophagectomy for esophageal cancer treatment. Currently, over-the-scope clip (OTSC) is an effective way to treat gastric tube perforation. Due to the lack of OTSCs, we invented an alternative method composed of a titanium clip and gastroscope. The aim of this study was to describe this novel endoscopic device in the treatment of gastric tube perforation. We used a titanium clip system to treat 4 male patients (range, 53 to 77 years with gastric tube perforation. After the location of the perforation was identified by gastroscope, a titanium endoscopic clip was used to close the perforation. Successful closure of the gastric tube perforation was achieved in three patients while in one patient this failed due to his refusal to undergo reoperation. No postoperative complication was found in the three patients whose perforations were closed and the patient who refused reoperation died due to the reoccurrence of his esophago-cardiac carcinoma. The endoscopic system composed of titanium clip and gastroscope proved to be an efficient and effective device in the treatment of the patients with gastric tube perforations.

## Background

Gastric tube perforation, which may be spontaneous or caused by other therapeutic measures, occurs as a very serious complication after esophagogastrostomy for esophageal cancer [1]. An ulcer, which could induce a perforation, is more prevalent in gastric tube patients than in the those with a normal healthy stomach. *Helicobacter pylori* infection, insufficient blood supply, gastric stasis and bile juice regurgitation play irrefutable roles in the formation of gastric tube ulcers and perforations [2]. Although these perforations are rare and often asymptomatic, severe septic conditions can be caused, which might ultimately result in a high morbidity and mortality [3], and even perforate the heart, pericardium and thoracic aorta [4,5].

Currently, surgery and conservation therapy are the main choices for the management of perforations [6]. The choice of the therapy mainly depends on the severity of the complication and the condition of patient. Conservation management with the placement of a nasogatric tube and antibiotic therapy might only be effective in those patients with small leakages or fistulas without septic complications [7]. Endoscopic surgical or radiological interventions are required for those patients with more severe leakage.

Advances in endoscopic closure devices have increased the therapeutic alternatives to primary surgery in these cases [8,9]. In 2009, Repici *et al*. [10] reported the application of the newly designed over-the-scope clip (OTSC) in the treatment of the patients with gastric or colonic bleeding and/or deep wall defect or perforation, and they proved OTSC to be an effective method for clinical treatment of perforations. Though our hospital lacks the OTSC device, we invented an alternative method composed of a titanium clip and gastroscope for gastric tube perforation treatment. Here, we describe our experience of the use of this newly invented clip system and evaluate its effectiveness in the treatment of gastric tube perforation.

## Case presentation

From January 2012 to December 2012, an end-to-side stapled esophageogastrostomy was performed in 339 patients with esophago-cardiac carcinoma. All the gastroesophageal anastomoses were completed by using the purse-string stapled anastomotic technique, and postoperative gastric tube perforations were experienced in four patients. The clinical diagnosis of anastomotic leak (AL) was identified by the presence of fever, leukocytosis and pululent drainage. The radiological diagnosis of AL was conducted by using meglucamine diatrizoate swallow study. All the patients were males and aged between 53 and 77 years. An overview of the patients’ age, gender, tumor pathology, lymph node dissection and stoma site are shown in Table 1.

**Table 1:** **Clinical characteristics of the four patients with gastric tube perforations**

**Case**	**Gender**	**Age**	**Site of tumor**	**Pathological types**	**TNM stage**	**Lymph node dissection (number)**	**Stoma site**
A	Male	58	Middle third of the esophagus	Squamous carcinoma	T3N0M0, IIa	14	Above the level of the aortic arch
B	Male	65	Middle third of the esophagus	Atypical hyperplasia (grade III)	T1N0M0, Ia	10	Above the level of the aortic arch
C	Male	53	Lower esophagus	Squamous carcinoma	T3N1bM0, IIIb	26	Above the level of the aortic arch
D	Male	77	Gastroesophageal junction region	Adenocarcinoma	T4N2M0, IIIc	12	Inferior to the level of the aortic arch

TNM stage depending on *TNM Classification of Malignant Tumours, 7* ^*th*^ *Edition*.

**Figure 1:**
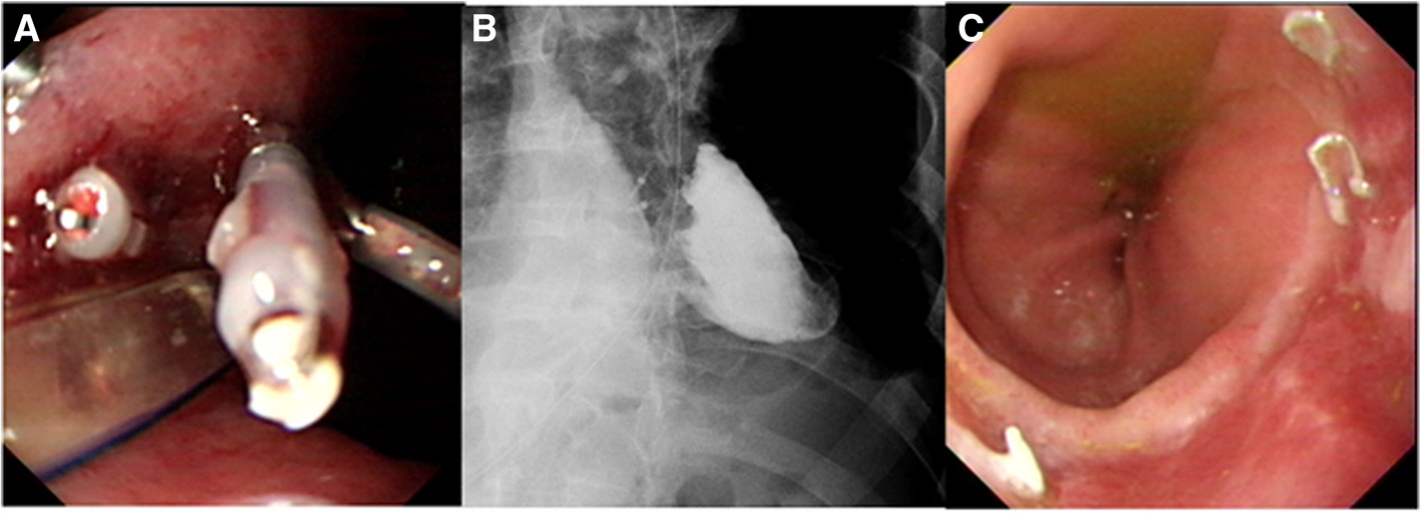
**Endoscopic clip closure of gastric tube perforation and follow-up. (A)** The perforation was closed by several clips. **(B)** Radiological gastrografin swallow study showed no residual leakage and two clips could be found on the film. **(C)** Gastroscopy was performed 6 months later and demonstrated a well-healed anastomotic stoma.

The procedure consisted of inserting the endoscope and loading the clipping device. After the patients were placed in a lateral position, the gastroscope (Olympus GIF-XQ 260 endoscope, Olympus Optical Inc, Tokyo, Japan) was inserted into the intrathoracic stomach and inflated with air. The gastric perforation could then be found with the help of the gastroscope and the confirmation of the perforation was achieved by normal saline injection into the thoracic cavity and the presence of normal saline in the stomach. After the perforation was identified, we sequentially closed the fistula from the ends to the middle by using endoclips (Olympus HX-600-135, Olympus Inc, Tokyo, Japan) (Figure 1A). The achievement of closure was confirmed by the absence of leakage of normal saline in the stomach after thoracic cavity saline injection. A postoperative meglucamine diatrizoate swallow study was carried out to double-check the endoscopic closure (Figure 1B). The clips would be seen to have fallen off into the gastrointestinal tract one by one (Figure 1C).

Case 1: a 58-year-old man was diagnosed with AL according to the clinical symptoms and result of meglucamine diatrizoate swallow study. Conventional treatments including enteral fasting and parenteral nutrition and intensified antibiotic therapy were initially administeredto the patient. Due to the lack of improvement in the infection, we performed the endoscopic procedure and found two perforations (4 mm and 7 mm, respectively) in the patient. These two perforations were then closed successfully by using the endoscopic clips. After the procedures, the leakage decreased immediately and recovery of the patient was confirmed by the increasein blood albumin level. The patient was given a liquid diet after a 13-day period and after 29 days the thoracic tube was extracted. Thirty-three days later, the patient was discharged from hospital with a good nutritional status (Table 2A).

**Table 2:** **Diagnosis and interventions for perforations**

**Case**	**Time of clinical diagnosis of AL**	**Time of radiological diagnosis of AL**	**Time of endoscopic closure; size of perforation**	**Time of liquid diet post-closure**	**Time of tube extraction post-closure**	**Post-closure stay**
A	7 days	12 days	59 days; 4 mm and 7 mm	13 days	29 days	33 days
B	19 days	NA	44 days; 5 mm	43 days	115 days	128 days
C	20 days	NA	First closure: 24 days; Secondary closure: 36 days; 3 mm	13 days	15 days	18 days
D	4 days	8 days	50 days; 5 mm,	42 days	47 days	50 days

AL: Anastomotic leakage; NA: Not applicable.

Case 2: a 65-year-old man underwent laparoscopic debridement and drainage of infected necrosis in an acute pancreatitis episode at 8 days after esophageal carcinoma resection. The patient was then required to fast, diagnostic peritoneal lavage was performed and broad-spectrum antibiotics administered. However, a sudden high fever and feculent chest drainage presented on the 19th day after the acute pancreatitis episode. A 5 mm gastric tube perforation then presented at 44 days after esophagetomy. The endoscopic procedure was performed to close the perforation. Despite his poor general health, the patient finally recovered and was discharged at 4 months after the endoscopic closure, by which time an improved nutritional status was observed (Table 2B).

Case 3: a 53-year-old man underwent radical surgery for esophageal carcinoma and was clinically diagnosed with a digestive system fistula with the presence of opacitas drainage at 20 days after surgery. Gastroscopy was performed 4 days later, and a 3-mm perforation was found. The endoscopic clip was then applied, with gastroscopic help, to close the perforation. Due to the presence of sustained muddy drainage, gastroscopy was performed again 12 days later and a residual leakage was found. The residual leakage was then closed by the endoscopic clip. The patient was started on oral liquids and the thoracic tube was extracted after 14 days (Table 2C).

Case 4: a 77-year-old man who had a history of myelodysplastic syndrome underwent radical surgery for cardia cancer. The patient was suspected as having an AL due to the presence of early postoperative opacitas drainage, fever and leukocytosis. The AL was further confirmed by gastrografin swallow study. Conventional treatments including enteral fasting and parenteral nutrition and intensified antibiotic therapy were administered to the patient for 6 weeks. However, the nutritional status of the patient deteriorated. The endoscopic procedure was performed in the patient and a 5-mm perforation was closed at 50 days post operation. A residual leakage was found by gastrografin swallow study but a second endoscopic clip was not performed due to refusal by the patient’s family. The patient took a long time to recovery and had lost 10 kg in weight at the time of discharge (Table 2D).

By 1 January 2014, the fourth patient had died due to tumor recurrence, but the other three patients were living and with a good nutritional status. In all three suvivors, a meglucamine diatrizoate swallow study was performed and no residual leakage was found.

### Discussion

In this study, we presented a case series on the treatments of post-esophagectomy gastric tube perforations by using a novel alternative method composed of titanium clip and gastroscope. A total of four patients were treated with an endoscopic procedure and a total of five perforations were closed with endoscopic clips. No postoperative complication was found in three patients whose perforation closure was achieved and the other patient died due to the reoccurrence of his esophago-cardiac carcinoma. Our results suggested that the endoscopic clip is an inexpensive, safe and tolerable method for the patient with post-esophagectomy gastric tube perforation. In addition, this endoscopic procedure also presented the advantages of easy operation and avoidance of surgery and long-term conventional treatment.

According to the previous study, self-expanding stents are currently the mainstay of endoscopic therapy for gastrointestinal perforations due to their excellent ability for treating even grade IV perforations [11]. However, it is difficult to close gastric tube perforations due to the irregularity of gastricconfiguration; furthermore, stent dislocation remains a common problem [12]. Also, stent therapy failureoccurred in approximately 15% of cases [13]. Recently, the OTSC endoscopic clipping device has become the new option for perforation treatment. The OTSC exhibited a high success rate, limited postoperative complications, and good long-term clinical outcomes [14]. Due to the lack of resources+, we have invented an alternative method composed of titanium clip and gastroscope. Hre, we used our system to close five perforations and the largest perforation size was 7 mm. Shin *et al*. reported that different types of clip have different attachment durations at the site of clip application [15]. Here, we chose the titanium clip and the results were encouraging. In addition, an appropriate *in vivo* environment is another important factor for successful endoscopic closure: if the patient presents with muscosal edema, the closure should be delayed.

Certain limitations were also present in our endoscopic system, as with other through-the-scope clips [16,17]. The efficacy of our system is restrained by their small wing span and the low compression force that the clips can withstand. In addition, we have no idea about size limitation of the perforation in our endoscopic system or the optimal time for performing the endoscopic intervention. Therefore, further studies are needed to optimize our system.

## Conclusions

In conclusion, herein we proposed a self-made simple endoscopic system to treat gastric tube perforation and it proved to be a safe, feasible and efficacious system for perforation closure if the OTSC system is unavailable.

## Consent

Written consents were provided by these four patients for all the material presented in this case series. A copy of written consent required by the Editor-in-Chief is available for review.

## Abbreviations

AL: Anastomotic leak
OTSC: Over-the-scope clip
